# Biting Midges (Diptera: Ceratopogonidae) as Vectors of Viruses

**DOI:** 10.3390/microorganisms11112706

**Published:** 2023-11-04

**Authors:** Helge Kampen, Doreen Werner

**Affiliations:** 1 Friedrich-Loeffler-Institut, Federal Research Institute for Animal Health, 17493 Greifswald, Germany; 2 Leibniz Centre for Agricultural Landscape Research, 15374 Muencheberg, Germany; doreen.werner@zalf.de

**Keywords:** *Culicoides*, vector, arbovirus, disease, distribution, epidemiology, transmission, microbiota, interaction

## Abstract

Biting midges of the genus *Culicoides* occur almost globally and can regionally and seasonally reach high abundances. Most species are hematophagous, feeding on all groups of vertebrates, including humans. In addition to being nuisance pests, they are able to transmit disease agents, with some viruses causing high morbidity and/or mortality in ruminants, horses and humans. Despite their impact on animal husbandry, public health and tourism, knowledge on the biology and ecology of culicoid biting midges and their interactions with ingested pathogens or symbiotic microorganisms is limited. Research is challenging due to unknown larval habitats, the insects’ tiny size, the inability to establish and breed most species in the laboratory and the laborious maintenance of colonies of the few species that can be reared in the laboratory. Consequently, the natural transmission of pathogens has experimentally been demonstrated for few species while, for others, only indirect evidence of vector potential exists. Most experimental data are available for *Culicoides sonorensis* and *C. nubeculosus*, the only species kept in western-world insectaries. This contribution gives an overview on important biting midge vectors, transmitted viruses, culicoid-borne viral diseases and their epidemiologies and summarizes the little knowledge on interactions between biting midges, their microflora and culicoid-borne arboviruses.

## 1. Introduction

Regionally and seasonally, blood-feeding biting midge species (Diptera: Ceratopogonidae) can be a serious nuisance to livestock and humans. Their aggressive biting behaviour, linked to regular high abundances, affects animal husbandry, human activities in agriculture and forestry and tourism in many areas of the world [1,2,3,4,5,6]. More importantly, numerous species of the ceratopogonid genus *Culicoides* are capable of transmitting viruses, protozoans and nematodes during feeding [7]. More than 50 viruses have been isolated from field-collected *Culicoides* [8], most notably bluetongue virus (BTV), epizootic haemorrhagic disease virus (EHDV), Schmallenberg virus (SBV), African horse sickness virus (AHSV) and Oropouche virus (OROV). BTV, EHDV and SBV are pathogenic to ruminants, and AHSV is pathogenic to equids, whereas OROV is the only known culicoid-borne virus linked to disease in humans. Outbreaks usually occur epidemically, cause considerable morbidity and mortality and lead to high economic losses [9,10,11,12,13]. As with most viral diseases, specific treatment is not possible, calling for other means of disease management, such as vaccination or vector control.

Although this contribution focuses on the above viruses, *Culicoides arakawae* transmits the protozoan *Leucocytozoon caulleryi*, another economically important biting midge-borne pathogen, causing huge problems in poultry farming in Asia [14].

Except for Australia, all continents inhabited by *Culicoides* species are affected by bluetongue, epizootic haemorrhagic disease, Schmallenberg disease, African horse sickness or Oropouche fever, although only bluetongue has been reported from each of the continents.

## 2. Bluetongue, Epizootic Haemorrhagic Disease, African Horse Sickness, Schmallenberg and Oropouche Viruses

### 2.1. Bluetongue Virus

BTV belongs to the genus *Orbivirus* of the family Reoviridae. It is an uncoated virus with a diameter of about 80 nm. Its genetic information is located on double-stranded RNA subdivided into ten linear segments of different lengths, which encode for seven structural proteins (VP1-7) and six non-structural proteins (NS1-5, NS3a) [15]. In addition to 24 ‘classical’ BTV serotypes, as determined by their VP2 gene products, several ‘atypical’ serotypes have been described, which are characterized by unusual features, such as avirulence or vector-independent transmission [15].

### 2.2. Epizootic Haemorrhagic Disease Virus

Belonging to the same virus family and genus, EHDV is closely related to BTV. Like BTV, it is non-enveloped and about 80 nm in diameter. Its seven known serotypes are differentiated according to the recognition specificity of neutralising antibodies towards the outer capsid protein VP2. Ten linear double-stranded RNA segments encode seven structural (VP1-VP7) and four non-structural proteins (NS1, NS2, NS3a, NS3b) [16].

### 2.3. African Horse Sickness Virus

Like BTV and EHDV, AHSV is a non-enveloped RNA-*Orbivirus*. It has a particle size of about 70 nm and ten double-stranded RNA segments, seven of which encode structural proteins (VP1-7,) and the remaining three encode four non-structural proteins (NS1-3, NS3a), with one RNA segment coding for two of those (NS3, NS3a). AHSV comes with nine serotypes, which are, again, determined by the highly variable outer capsid protein VP2 [17,18].

### 2.4. Schmallenberg Virus

SBV is a virus of 80-120 nm diameter, assigned to the Simbu serogroup of the genus *Orthobunyavirus*, family Peribunyaviridae. Its negative-sense single-stranded RNA genome consists of three segments (L, M, S) that encode four structural (RNA-dependent RNA polymerase, N, Gn, Gc) and two non-structural proteins (NSs, NSm) [19]. Its origin is unclear, but it has been suggested to be the product of a reassortment, combining the M-segment of Sathuperi virus and the L- and S-segments of Shamonda virus [20]. By contrast, a more recent study based on comparative genetic and serologic analyses concluded that SBV might be a possible ancestor of Shamonda virus, in which a reassortment of the L- and S-segments of SBV and of the M-segment of an unknown virus had taken place [21].

### 2.5. Oropouche Virus

The systematic assignment, structure and organization of OROV are exactly as for SBV. Its diameter is about 90 nm [22,23].

## 3. Pathology of Bluetongue, Epizootic Haemorrhagic Disease, African Horse Sickness, Schmallenberg Disease and Oropouche Fever

### 3.1. Bluetongue

Ruminants infected with BTV often become conspicuous by lameness, reluctance to move and emaciation, which are caused by muscle and claw inflammation. Fever outbreaks, circulatory disorders, increased salivary secretion, hyperaemia, oedema and ulcer formation in the head area, severe breathing difficulties and skeletal muscle changes are also typical symptoms. The origin for the name of the disease comes from the bluish or blue-reddish discolouration that occasionally occurs at the oral mucosa and on the tongue due to cyanosis. Frequently, petechial haemorrhages and inflammation occur on the teats. In diseased dams (female mother animals), stillbirths and malformations of the foetuses can occur [24,25].

### 3.2. Epizootic Haemorrhagic Disease

Depending on the virus serotype, clinical signs of infection are particularly severe in cervids and cattle [26]. In white-tailed deer (*Odocoileus virginianus*), hyperacute, acute and chronic forms occur, with estimated mortality rates of 20% [27], sometimes reaching 90% [28], in the hyperacute and acute forms. The hyperacute form presents with fever, respiratory distress, anorexia, weakness and oedema of the head and neck. Swelling of the tongue and conjunctivae can occur. Infected animals often die within days after infection. In the acute (classical) form, these symptoms can be accompanied by ulcers and haemorrhages of the skin, heart, and gastrointestinal tract. In the chronic form, animals can recover after several weeks but often become lame and lose their hooves, causing them to crawl on their knees [16,26]. Clinical manifestations in cattle are usually less severe, but similar symptoms to those in deer have occasionally been observed, as well as abortions, foetal malformations and stillbirths [16,26]. Disease symptoms have also been reported from mule (*Equus caballus* × *asinus*), black-tailed deer (*Odocoileus hemionus*), bighorn sheep (*Ovis canadensis*), yak (*Bos mutus*), elk (*Alces alces*), brocket deer (*Mazama* spec.) and pronghorn antelope (*Antilocapra americana*), whereas seropositivity without clinical signs has been found in fallow deer (*Dama dama*), wapiti (*Cervus canadensis*), bison (*Bos bison*), sheep (*Ovis gmelini*), goat (*Capra aegagrus*), red deer (*Cervus elaphus*) and roe deer (*Capreolus capreolus*) [26].

### 3.3. African Horse Sickness

AHSV exclusively affects equids, with the disease presenting with fever, oedema, sweating, breathing difficulties, coughing attacks, frothy discharge from the nostrils, swellings in the head area, haemorrhages and colic [29]. Most infected, non-immunised animals are doomed to die, frequently resulting in fatality rates of 80–90% [30].

### 3.4. Schmallenberg Disease

In adult ruminants, SBV infection usually remains asymptomatic. Acute infections can cause mild symptoms, such as fever, diarrhoea or milk decline. Transplacental infection during a sensitive period of pregnancy, however, leads to severe malformations (joint stiffness, tendon shortening, torticollis, hydranencephaly, hydrocephalus), stillbirths, preterm delivery and mummification in embryos and foetuses [31].

### 3.5. Oropouche Fever

Oropouche fever, occurring in Central and South America [22], is the only *Culicoides*-borne human disease known. It usually strikes at high prevalences (up to 20%), affecting thousands of citizens in a given region at the same time and causing considerable social and economic impacts [12]. The infection commonly manifests with acute febrile illness with influenza-like symptoms, such as dizziness, chills, headache, myalgia, arthralgia and anorexia [32,33]. Typically, the incubation period lasts about 4–8 days and the period with clinical signs another 2–7 days [12,32]. About two-thirds of the patients experience a second milder episode after the first one, but—despite sporadic cases of meningitis [34,35]—recovery is complete, and no fatalities have been reported [32].

## 4. Disease Epidemiology

### 4.1. Bluetongue

Bluetongue is widely distributed in Africa but is also common in North America, northern South America, Europe, the Middle East, South and Southeast Asia, Oceania and Australia. The epidemiological systems (episystems) in the various geographic areas differ, depending on BTV serotypes and vector species [36].

Bluetongue has a long history in Africa. In southern Africa, typical bluetongue disease symptoms have been known from sheep since the late 18th century, but the first detailed scientific descriptions were provided only at the turn of the 19th to the 20th century [37,38]. Disease incidences or infection prevalences from that time are not available, but ruminant game, particularly antelope, meant to be exported between 1997 and 2000, showed seroprevalences against BTV of up to 45% [39].

By the mid-20th century, the disease also emerged in North America, where it first became known as a disease of sheep called “soremuzzle” [40]. The causative virus was identified in the United States in 1952 [41].

In the late 1950s and the early 1960s, bluetongue was reported for the first time from South Asia. Outbreaks occurred in 1958 and 1964 in sheep populations of Pakistan and India, respectively, with more later occurring in several regions of the Indian subcontinent. BTV is now considered endemic in both Pakistan and India [42]. To the east, cases were registered in China in the late 1970s, in Indonesia and Malaysia in the 1980s and in Japan in the 1990s [43].

In Europe, bluetongue probably emerged by 1924 and resurged during successive years and decades in Cyprus, with a major outbreak in 1943 [44]. Epidemics in continental Europe were observed no earlier than 1956 on the Iberian Peninsula, where 180,000 sheep were killed by 1957 [45]. After 1998, outbreaks increased in number in mainland Europe and were registered in much further northern areas than before. This development was consistent with a northward expansion of the distribution range of *Culicoides imicola*, an Afro-Asian biting midge representing the world’s most important vector species, which was probably facilitated by climate warming [46].

From 2006 to 2009, an unprecedented epidemic of BTV serotype 8 (BTV-8) occurred in Central and Northern Europe [47]. In the first year of the outbreak, infections were diagnosed in more than 2000 ruminant holdings in the Netherlands, Belgium, Luxembourg, Germany and France. The disease spread to additional European countries in the following year, involving almost 60,000 holdings. In 2008, more than 27,000 holdings were affected [48], but large-scale vaccination quickly took effect and caused a drastic decline in case figures by 2009 [49].

Apparently, BTV is regularly introduced to Australia from Asia by wind-borne infected biting midges [50,51]. The first detection of the virus in Australia dates from 1975 [52], and multiple virus serotypes have since been detected there [53]. For reasons not clear, clinical disease, however, has not yet become known [54]. Wild ruminants (i.e., cervids) are the natural reservoirs of BTV in Australia [55].

Effective vaccines against several BTV serotypes exist and have been used in different parts of the world [56]. After express development and admission of a BTV-8 vaccine and its immediate administration, bluetongue spread was controlled in Germany, for example, within less than two years [49].

### 4.2. Epizootic Haemorrhagic Disease

Attributed to an outbreak among white-tailed deer in 1955, epizootic haemorrhagic disease was first described in New Jersey, USA, followed by the isolation of the causative virus [57]. During the next few decades, it was identified on all continents, except for Europe, and is now considered endemic in parts of North America, Australia and some Asian and African countries [58]. Severe outbreaks in cattle were reported from Japan in 1959 (more than 4000 deaths) and 1997 [59,60], but they have increased in number recently, such as in the USA, Asia, the Middle East and North Africa [16,58]. Coincidentally, an expansion of outbreak areas has been observed: after the turn of the millennium, epizootics occurred in Mediterranean countries, such as Israel, Morocco, Turkey, Egypt and Tunisia [16,26], and in late 2022, the disease was first diagnosed on the European continent, in Italy (Sicily, Sardinia) and Spain (Andalusia) [59]. The Italian virus strains were genetically identical to the strain responsible for an outbreak in Tunisia in 2021, suggesting a spill-over from northern Africa to southern Europe [61,62]. Reasons for the increase in disease severity and the geographic spread are not yet fully understood but have been suggested to be linked to newly introduced serotypes by animal movement or wind drift as well as climate change [16,26,58].

Live-attenuated or inactivated vaccines are available only against EHDV-1, EHDV-2 and EHDV-6 [63].

### 4.3. African Horse Sickness

Areas enzootic for African horse sickness are widely distributed in sub-Saharan Africa, predominantly southern Africa, but disease outbreaks have also been reported from North Africa, the Middle East, the Indian subcontinent and southern Europe [17,29]. In the 17th century, South Africa faced an outbreak during which about 70,000 animals (some 40%) of the local horse population died, while in Namibia, almost 1000 horses succumbed to the disease [30]. Between 1959 and 1961, 300,000 equids died of AHSV infections in the Middle East and the Indian subcontinent [29]. Outbreaks were also observed in 1966 and from 1987 to 1989 on the Iberian Peninsula, with about 3000 horses being slaughtered during the latter outbreak [30]. In 2020, the first outbreak was reported from Southeast Asia (Thailand). Immediate vaccination prevented high mortality, but a total of about 2700 horses became infected [64]. Wild equids (i.e., zebra and African donkey) are the reservoirs of AHSV [65].

Live-attenuated vaccines are available for African horse sickness. The currently used vaccines have risks and shortcomings, but promising candidates are in the pipeline [17].

### 4.4. Schmallenberg Disease

In 2011, SBV emerged in Central Europe as a completely novel virus [66,67]. It was detected in the context of a disease outbreak in Germany, the Netherlands and Belgium, during which more than 90% and regionally close to 100% of the cattle tested seropositive for infection [68,69]. Until 2012, SBV spread to numerous European countries, with infections reported from nearly 6000 holdings [67,70]. Outside Europe, antibodies against SBV have been demonstrated in ruminants, including cattle, that delivered stillborn and malformed calves in China and various parts of Africa [71,72,73]. As with BTV, wild ruminants represent natural reservoirs of SBV [55]. Existing vaccines against Schmallenberg disease are based on inactivated whole virus and are used restrictively; marker vaccines are being developed [74,75].

### 4.5. Oropouche Fever

Oropouche fever is restricted to Central and South America, with a recent outbreak having occurred in the Caribbean [23]. Case incidences average about 30%, and the total number of cases between 1961 and 2007 was estimated at about 380,000 [22]. Mammals, including sloths and non-human primates, as well as wild birds, are thought to serve as natural reservoirs [30]. Efforts to develop vaccines against Oropouche fever have not been successful so far but are ongoing [23].

## 5. Biting Midges

### 5.1. Systematics and Biology

According to the most recent “Catalog of the Biting Midges of the World”, as compiled by Borkent and Dominiak [76], there are 6206 extant valid species of the dipteran family Ceratopogonidae worldwide, placed in three subfamilies (Leptoconopinae, Forcipomyiinae and Ceratopogoninae) with 112 genera. Among these, the genus *Culicoides* is the most notorious; many of its 1347 species are hematophagous and potential vectors of disease agents.

Biting midges are tiny culicomorph insects, usually measuring 1 to 2.5 mm in length [77]. Their larvae develop in all types of aquatic, semiaquatic and terrestrial humid habitats, with many species using the entire range of these [78]. Larvae are so diverse regarding habitus, behaviour and ecology, and difficult to study that they often cannot be assigned to their conspecific adults [79].

Adults can be significant pollinators [80], and all developmental stages play important roles in the food web. In some genera (e.g., *Dasyhelea*), the adults of most species live exclusively on nectar in both sexes, whereas in others (e.g., *Culicoides*, *Forcipomyia* and *Atrichopogon*), the females usually need a proteinaceous meal to produce eggs. Depending on the species, they feed on the haemolymph of invertebrates, including various groups of arthropods, or on the blood of vertebrates, including mammals, birds, reptiles, amphibians and even fish, using their piercing mouthparts to penetrate the host‘s tegument or skin [76,78]. Still, other species are predaceous, and some are pollinivorous.

### 5.2. Biting Midge Vectors of BTV, EHDV, AHSV, SBV and OROV

The natural transmission of BTV by *Culicoides* biting midges was experimentally demonstrated by Du Toit in South Africa in the 1940s [81]. By injecting emulsified field-collected biting midges, most likely *C. imicola*, into horses, Du Toit could also show that the insects carried the agents of African horse sickness. Natural transmission of AHSV by culicoids during blood feeding was demonstrated much later by Wetzel et al. [82].

*C. imicola* is the most important biting midge vector worldwide. It has been reported throughout Africa (except for the Sahara), in the Near and Middle East, parts of South and Southeast Asia, and southern Europe [83], where it is responsible for the majority of bluetongue and African horse sickness cases [84,85]. Another important vector of BTV and AHSV in Africa south of the Sahara is *C. bolitinos,* a closely related species of the Imicola Complex [86,87].

Although *C. imicola* had been found in Cyprus in the early 1970s [88], the first records of this species from mainland Europe (southern Spain) stem from 1982 [89,90]. Early introductions of BTV and AHSV to Cyprus and the Iberian Peninsula were considered to have been caused by single wind-borne infected biting midges from North Africa [91,92,93]. However, European-native species belonging to the Obsoletus Group were suspected to possess vector competence for BTV by the late 1970s after the isolation of BTV from unengorged, parous females during an outbreak in Cyprus [94]. Further indications that *C. obsoletus* and *C. pulicaris*, or species of the complexes they are eponymous for, might play a role in BTV transmission in southern Europe came in the early 2000s [95,96]. Similar findings existed from the late 1980s that species other than *C. imicola* might be responsible for AHSV transmission in southern Europe [97]. Still, bluetongue occurrence matched quite well with the geographic distribution of *C. imicola* until the early 2000s. The most northern European region where *C. imicola* has been detected (one specimen) is Ticino, southern Switzerland [98].

The final demonstration that biting midge species native to Europe are competent and efficient vectors of BTV came in 2006, when bluetongue disease broke out in Central and Northern Europe [41]. During that epidemic, comprehensive biting midge monitoring was carried out in numerous affected countries, with Obsoletus Group species being, by far, the most frequent ceratopogonid species collected, followed by Pulicaris Group species [99,100,101,102]. *C. imicola* was not encountered. The dominance of Obsoletus Complex species in the European culicoid fauna was later confirmed in a multinational study along a transect from northern Sweden to southern Spain, with 83% of the collected biting midges belonging to this group and 12% belonging to the Pulicaris Group [103]. Triggered by the European 2006 outbreak, several studies were published trying to genetically differentiate the isomorphic or morphologically similar species of the Obsoletus and Pulicaris Groups to be able to identify vector species [104,105,106,107].

In North America, *C. variipennis* was demonstrated to be vector-competent for BTV in 1963 [108]. Based on data from isoenzyme analyses, this species was later subdivided into several forms or subspecies, with *C. variipennis sonorensis*, later ranked as a true species, *C. sonorensis*, apparently being the major North American BTV vector [109]. Evidence for further North American *Culicoides* species playing noteworthy roles as BTV vectors is missing [110]. In Central and South America, *C. insignis* is the most important vector, whereas in Australia, despite inefficient transmission but due to its wide distribution and high abundance, *C. brevitarsis* (Imicola Complex) appears to be the major vector [80]. In addition to *C. imicola*, numerous endemic and some cosmopolitan culicoid species are considered potential BTV vectors in Asia [80].

Epidemiology and field data suggest that AHSV is basically transmitted by the same *Culicoides* species as BTV, and these are also assumed to be the culicoid vectors of EHDV [90]. The major EHDV vector in North America is again thought to be *C. sonorensis*, with other species, such as *C. insignis*, being vectors outside the distribution area of *C. sonorensis* [17,111,112]. *C. imicola, C. bolitinos* and *C. schultzei* are the main implicated vector species in Africa, those of the Obsoletus and Pulicaris Groups in Europe, *C. imicola and C. oxystoma* in Asia and *C. brevitarsis* in Australia [17].

In Europe, SBV appears to have the same vectors as BTV, primarily species of the Obsoletus Group [113,114,115,116]. However, experimental vector competence studies are rare [117].

OROV transmission potential has been demonstrated in the laboratory for *C. paraensis* [118], *C. sonorensis* and the mosquito *Culex quinquefasciatus* [119,120]. Due to efficient transmission, its anthropophilic biting behaviour and demonstrated high abundances in affected regions, *C. paraensis* is considered the major vector in the urban transmission cycle [90]. Its distribution is not confined to South America but includes considerable parts of the USA [121].

Major *Culicoides* species and evidence for them to act as virus vectors in different parts of the world are compiled in Table 1.

## 6. Biting Midge Laboratory Studies

Infection studies are essential to examine pathogen–vector interactions, including the determination of vector competence and efficiency under given conditions. Detailed knowledge on these aspects allows for investigations into the biology, ecology and spatiotemporal distribution of vector species and helps in our understanding of the epidemiology of culicoid-borne diseases and in designing vector management plans.

Although culicoids were recognized as vectors of disease agents decades ago and were extensively studied on other continents, species native to Central Europe have long been considered merely as nuisance pests. Only with the 2006 outbreak of bluetongue disease did biting midge vector research increase in many European countries. In the wake of pertinent efforts, culicoid systematics and species differentiation, particularly within the Obsoletus and Pulicaris Groups, have been identified as pivotal and unresolved issues. Recent studies using DNA sequencing and MALDI-TOF technologies have suggested that these groups consisted of more genetic variants, and probably species, than previously thought and that a thorough revision of the genus *Culicoides* is necessary [143,144,145,146,147].

Few biting midge species can be reared in the laboratory, such as *C. sonorensis*, the major vector of BTV in North America, *C. nubeculosus*, a European species of negligible importance as a potential BTV vector, and *C. arakawae*, an Asian vector of BTV and *L. caulleryi* [148,149,150], but neither *C. imicola* nor European species considered important putative virus vectors. This immensely impedes conducting controlled experiments with a wide range of species, for example, on vector competence or pathogen–host interactions. To compensate for that, many studies use field-collected biting midges. A major obstacle to that approach is that it is often not known at the start of the experiment which species are involved and whether sufficient numbers of the desired, or at least of the same, species are included for adequate statistical power. Genetic identification of the specimens is not possible before the experiments, and morphological identification, which is possible only on part of the species, will require short-term anaesthesia (e.g., by CO_2_ or cold exposure) that causes the death of many of them. An unsatisfactory compromise is to collect the biting midges at a place and time that the desired species is known to be most abundant. In any case, species identification must be performed genetically after the end of the study. A second critical point in such studies is that the field-collected biting midges, which are extremely sensitive to laboratory conditions, must feed on blood and survive for a certain period of time. In vector competence studies, the midges ideally must feed twice, once initially on infected blood or an infected host and again on clean blood or a naïve host, to demonstrate virus transmission [124,151]. This turns out to be critical, and it can be challenging to make the biting midges feed only once. If that is successful, they can at least be examined after a given (extrinsic) incubation period to determine the oral susceptibility to virus infection by virus titration or quantitative PCR, preferably on separated heads with salivary glands attached to deduce vector competence and transmission indices [117,126,152,153]. Still another infection technique is intrathoracic injection [123,129], which might be appropriate to examine host–virus interactions but is of limited value for vector competence studies since it circumvents the insect’s midgut barriers [154]. Most of the little knowledge available on biting midge–virus interactions has been obtained from oral and intrathoracical infection studies and insect cell culture studies.

Virus replication in vector-competent biting midges is temperature-dependent in the modest number of infection experiments that have been conducted. At 25 °C, *C. sonorensis* is able to transmit BTV from seven days post-infection onwards [155]. Although Carpenter et al. [156] determined a relatively consistent minimum replication temperature of 11–13 °C across different orbiviruses and different *Culicoides* vector species, Wellby et al. [157] and Mullens et al. [158] reported AHSV and BTV to stop replication below 15 °C. However, latent virus seems to persist at low temperatures and can continue to replicate when temperatures increase again [157,158].

## 7. Effects of the Biting Midge Vector on the Virus

The vector competence of a hematophagous arthropod for a pathogen is primarily innate and genetically determined, depending on arthropod species, strain or population and pathogen species and strain [159,160]. It is a product of evolutionary adaptation, during which the pathogen develops mechanisms (e.g., metabolic pathways, cell attachment and entry and immune modulation or evasion) to infect the arthropod, replicate or continue development in its cells, organs or body cavity, evade its immune defence and enter the salivary ducts or migrate into the proboscis to be transmitted to a vertebrate host during blood feeding. In addition to genetic factors, the vector competence of an insect can be modified by stress factors acting on the larval stage (e.g., temperature, inter- and intraspecific competition, food supply) and, as discovered only recently, the insect’s microbiota [161].

Vector competence is, thus, not general for a hematophagous arthropod species but usually works only with a limited number of pathogens. Exceptionally, vector competence for a certain pathogen is given in an arthropod species that has never encountered that pathogen before. In these cases, the pathogen is likely to have co-evolved with, and adapted to, a closely related arthropod species or to have accidentally developed surface epitopes or mechanisms that allow for development and transmission [162].

**Figure 1:**
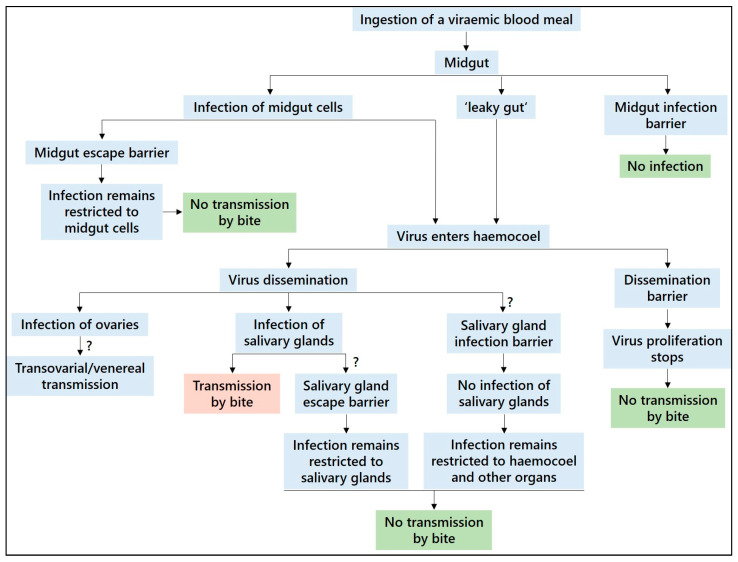
Migration of arboviruses through insect vectors and developmental barriers. Question marks denote barriers that have not yet been demonstrated in biting midges. Adapted from Hardy et al. [170] and Mellor et al. [90].

Some viruses may be passed on venereally or transovarially by an arthropod (Figure 1), and, in these cases, the pathogen has to reach the cells of the reproductive organs instead of, or in addition to, the salivary glands. At the least, transovarially infected arthropods are thought to have disseminated infections and are also able to transmit the pathogen via a bite during the first bloodmeal [163]. There is no evidence that BTV, EHDV, AHSV and OROV are transmitted vertically [90,164], and the findings of SBV in nulliparous *C. obsoletus/scoticus* complex and *C. punctatus* specimens [145], indicating transovarial transmission, are yet to be confirmed.

Apparently, the migration of the various biting midge-borne viruses through their insect vector is similar and does not differ basically from that of other arboviruses through mosquitoes or other hematophagous insects. In the case of biting midges as hosts, most details are known from studies on BTV and AHSV, whereas few studies exist on EHDV, SBV and OROV.

After ingestion of an infectious blood meal by the biting midge, BTV will enter the midgut where the virus titre decreases, sometimes to below detection limits, caused by both inactivation through digestive enzymes and the anal excretion of viral particles. Studies have shown that a minimum viral concentration exceeding a certain threshold in the blood meal is necessary for successful infection of the midgut epithelial cells and that the concentration of virus in the blood determines the infection rate [8,165,166].

Having invaded the mesenteronal cells, the virus starts to replicate, after which it is released into the haemocoel where it may infect secondary organs, such as fat body, muscle, Malpighian tubules, neural system, heart tissue, salivary glands and reproductive organs to add more replication cycles, thereby successively proliferating through the insect’s body [167]. The tissues and organs infected can vary, depending on the virus, host species and strain [90,168]. With respect to the dissemination of BTV into the salivary glands of *C. sonorensis*, Fu et al. [122] demonstrated that fat body, ganglia and salivary glands became infected subsequently, but no replication took place in the hindgut, muscle, Malpighian tubule and ovarian cells.

It is not clear whether the salivary glands are invaded from the haemocoel or from neural tissue, but both pathways seem possible. Within 3 days after infection, virus can be demonstrated in neural cells, suggesting that these can be used for dissemination. Once entering the acinar cells of the salivary glands, virus replication takes place again, with resulting viral particles being released into the terminal acini from where they can be transmitted by bite with the vector’s saliva [169]. Foster et al. [108] observed that the bite of a single infected biting midge is sufficient to infect a susceptible sheep.

While proliferating, the virus titre will increase in the biting midge until a plateau is reached [8,168]. For mosquitoes, innate mechanisms are hypothesized to control viral titres to prevent pathological effects of the virus [170]. In contrast to mammalian cells, which are heavily damaged by BTV exocytosis, viral release from insect cells appears to be gentle, happening exclusively by membrane budding [169].

Viruses that are not able to pass through an arthropod (i.e., for which the arthropod is vector-incompetent) are confronted with various barriers (Figure 1), preventing infection or the continuation of dissemination: midgut infection barrier, midgut escape barrier, dissemination barrier, salivary gland infection barrier and salivary gland escape barrier. In contrast to other groups of hematophagous arthropods, such as mosquitoes, the latter two barriers have not been demonstrated in culicoids [90,122], notwithstanding McGregor et al. [120] discussing a salivary gland barrier as the reason for the comparatively low experimental transmission potential of *C. sonorensis* for OROV.

As shown by various infection studies with mosquitoes and biting midges, including thoracic injection, where virus replication and dissemination take place without passage through the midgut epithelium, the mesenteron is the most important infection barrier in hematophagous insects. However, according to studies with *C. nubeculosus* and *C. sonorensis*, the peritrophic membrane is not able to prevent infection of the midgut cells. In *C. nubeculosus*, it begins to form at 5 h after blood meal ingestion [171], whereas in *C. sonorensis*, this happens only after 1 day [172]. In contrast, by 6 h after the infectious blood meal, virus particles can be associated with the microvilli of the midgut cells in *C. sonorensis* and having entered the first midgut cells (i.e., already during the time of peritrophic membrane formation). Predominantly from day 1 to day 4 after blood feeding, maturing virus particles are released from the epithelial cells through the plasma membrane into the extracellular spaces below the basal lamina. Only at 2 days after blood ingestion, a continual, although variably thick, peritrophic membrane is visible, while the blood volume in the midgut lumen is significantly diminished [172]. Blood digestion is complete after 4 days in virus-fed *C. sonorensis* but takes only 2 days in non-infected control specimens, suggesting that infection interferes with blood digestion [171,172]. The peritrophic membrane starts to degenerate in *C. sonorensis* by day 3 after the infectious blood meal [172].

Rather than the peritrophic membrane, processes directly connected to the midgut cells, such as attachment, penetration, uncoating and replication, are steps at this level that facilitate or inhibit infection. Among other factors, refractoriness to infection may be due to missing or non-matching midgut cell receptors for virus entry. As known from cell culture studies, BTV, for example, enters insect cells via clathrin-mediated endocytosis [173]. A detailed description of cell entry and virus replication was provided by Mertens et al. [174].

In vector-competent biting midges, the midgut escape barrier is passed within 2 days after infection, and virus appears in the salivary glands after 5 to 7 days at 25 °C [122,156,167].

In studies with *C. sonorensis*, subpopulations were identified, which developed only low virus titres and did not transmit BTV or AHSV. It could be demonstrated that the viruses remained restricted to the midgut cells of these biting midges and were not released into their haemocoel. A virus titre of <10^2.5^ TCID_50_/biting midge is considered to be linked to a midgut escape barrier, whereas a higher titre is presumed to indicate infection of the salivary glands and vector competence [8,122,132].

Both in the host cell and in the haemolymph, the virus is exposed to the antiviral mechanisms of the host, which contribute to the various developmental barriers. Although innate and not adaptive, the insect’s immune system is highly complex and efficient [175]. Feeding alone causes an upregulation in the expression of genes involved in the innate immune system [176]. According to the occurrence of genes and DNA sequences in the transcriptome and gene databases of *C. sonorensis* that are orthologous to functionally known genes of mosquitoes, the antiviral defence in the midgut epithelia has been suggested to consist of JAK-STAT (Janus kinase-signal transducers and activators of transcription) and siRNA pathway elements [173]. The siRNA pathway members identified in *C. sonorensis* have also been shown to be responsible for reductions in virus titres in cell culture studies [177]. Once virus particles have been released into the haemocoel, they are confronted with haemocytes, humoral antimicrobial peptides (AMPs) produced by Toll and Imd (immune deficiency) pathways, prophenoloxidases catalysing melanization and complement-like pathways [173], which are the major elements of the dissemination barrier. In some biting midges, virus concentrates in the fat body [8], which is a highly immunoactive organ in insects [178].

These barriers, most importantly the midgut barriers, might be disabled by stress factors acting on the arthropod’s larva, such as temperature variation, overcrowding and competition, resource limitation, predation and pesticide application, and lead to a modification of vector competence or even render a vector-incompetent arthropod vector competent [90]. Particularly, temperature can impact arbovirus transmission: at elevated larval rearing temperatures, the transition of a virus from midgut lumen to the haemocoel is facilitated via stress-linked leaks in the midgut epithelium (“leaky gut” phenomenon), allowing for an intercellular passage (Figure 1) [179]. This situation is comparable to an artificial parenteral infection by intrathoracic injection, whereby the midgut barriers are circumvented. *C. nubeculosus*, for example, which is generally known to possess a midgut infection barrier to BTV and AHSV, shows increasing infection rates with increasing developmental temperature, producing high virus titres [180,181].

## 8. Effects of Virus Infection on the Biting Midge Vector

No information exists on beneficial or adverse effects of BTV, EHDV, AHSV, SBV or OROV infection on the development and fitness of their *Culicoides* vectors, although, again, studies with mosquitoes and mosquito-borne viruses suggest so [182].

## 9. *Culicoides* Microbiota and Its Interaction with Culicoid-Borne Viruses Pathogenic to Vertebrates

Studies on the microbiota of *Culicoides* biting midges are scarce, mainly restricted to the bacterial midgut fauna, and differentiation, in the best case, to the genus level [183,184,185,186]. A few studies exist on the virome of *Culicoides* species [187,188,189,190]. Both groups of studies do not consider biotic interactions or correlate the midgut fauna with pathogen infection of the insect host, although an impact of bacteria and viruses must be considered likely based on studies with mosquitoes [191]. Only one study [192] investigated the biota of biting midges in the absence and presence of a vertebrate-pathogenic virus, SBV. The authors compared the bacterial midgut fauna of *C. nubeculosus* and *C. sonorensis* from laboratory colonies and found Protobacteria, Alphaprotobacteria, Acetobacterales, Acetobacteraceae and the genus *Asaia* to constitute the major part of the microbial fauna in *C. nubeculosus*, with the genus *Asaia* being predominant in untreated biting midge samples. By contrast, the *C. sonoresis* midgut fauna presented a more balanced mixture of partly the same and other bacterial taxa. Antibiotic treatment changed the midgut fauna completely and reduced *Asaia* in favour of *Delftia* bacteria. It was also associated with an increased infection rate of *C. nubeculosus* after feeding on SBV-spiked blood. Differentiation according to bacterial species was not provided in that study.

A couple of studies have focused on the presence of endosymbiotic *Wolbachia*, *Cardinium* and *Rickettsia* bacteria in *Culicoides* species. Nakamura et al. [193], for example, demonstrated *Cardinium* in 4 and *Wolbachia* in 1 out of 25 *Culicoides* species tested from Japan, while Mee et al. [194] found *Cardinium* in 19 and *Wolbachia* in 9 out of 20 *Culicoides* species in southeastern Australia. 16S rDNA and gyraseB gene sequences of all *Cardinium* clustered together in a new Candidatus *Cardinium hertigii* group (named group C), which is separate from *Cardinium* sequences known from other host families. Closely related *Cardinium hertigii* group C sequences were detected in *C. imicola, C. oxystoma* and *C. schultzei* group specimens from Israel [195]. In *C. imicola*, they could only be localized in the ovaries, including oocytes, pointing to a vertical mode of transmission. The first detection of *Wolbachia* (A and B supergroup strains) in *Culicoides* (several taxa, including *C. imicola, C. obsoletus* and *C. pulicaris* s.l.) in Europe was reported from Spain [196]. In the same study, *Cardinium* of the C group was detected. Based on finding *Cardinium* in *C. pulicaris* and *C. punctatus*, but not in the Obsoletus Group in the UK, Lewis et al. [197] concluded that these symbionts do not affect vector competence. *Rickettsia* of the Torix group were identified in 11 out of 29 *Culicoides* species collected in various areas of Europe [180]. Sequence analysis of rickettsial contigs from *C. newsteadi* revealed genomic features that could impact vector competence [198]. In all studies, *Cardinium* and *Rickettsia* prevalences were generally high. Similar to symbionts of culicids [199], symbionts of ceratopogonids might be exploited, if needs be, after genetic modification, to impair *Culicoides* reproduction and vector competence to reduce the risk of biting midge-borne disease.

Although pertinent data from *Culicoides* biting midges are virtually lacking, there is evidence from mosquitoes that species diversity and abundance of the microflora might influence the permissiveness or refractoriness of the midgut epithelia of hematophagous insects by modifying the immune status, thus affecting vector competence [200]. As shown for *Ae. aegypti* females cleared of *Serratia odorifera*, an omnipresent gut commensal of larvae and adults of this mosquito species, co-infection with *S. odorifera* and dengue virus serotype 2 (DENV-2) increased the viral susceptibility significantly, as compared to females only fed with DENV-2 or females co-fed with DENV-2 and *Microbacterium oxydans* [201].

## 10. Conclusions

As with other vector-borne diseases, culicoid-borne disease management can work only as an integrated approach. In view of largely missing or unsatisfactory treatments and vaccines, organizational, structural and vector control measures should be developed. For the latter, more research is necessary, both in the field and in the laboratory. Little is known regarding the impact of the biting midge’s biota on the biological traits of the midge itself, such as fitness, fecundity and vector competence, as well as arbovirus infection and transmission, by the biting midge. The relevance of the biota for the physiological processes in an organism’s body, including that of a vector, has only recently been recognized [202,203], and its composition and implications for an ectothermic organism are probably temperature-dependent and will change with climate warming [204,205,206]. Novel and advanced molecular genetic techniques will help provide better insight into this research area.

## Figures and Tables

**Table 1:** Demonstration of, or clues to, *Culicoides* species to act as virus vectors.

Culicoides Species	Primary Geographic Distribution	Virus	Quality of Virus Demonstration	References
C. sonorensis	North America	BTV	Experimental transmission after oral infection	[122]
C. sonorensis	North America	BTV	Virus dissemination ^a^	[117]
C. insignis	Central America, Carribbean	BTV	Indirect experimental transmission ^b^	[123]
C. obsoletus	Asia-Africa	BTV	Virus isolation ^c^	[94]
C. bolitinos	South Africa	BTV	Virus dissemination ^a^	[124]
C. obsoletus s.l.	Europe	BTV	PCR detection ^d^	[96]
C. obsoletus	Europe	BTV	PCR detection ^d^	[125]
C. scoticus	Europe	BTV	Virus replication after oral infection	[126]
C. chiopterus	Europe	BTV	PCR detection ^c^	[115]
C. pulicaris	Europe	BTV	PCR detection ^c^	[95]
C. dewulfi	Europe	BTV	PCR detection ^d^	[127]
C. achrayi	Europe	BTV	PCR detection ^d^	[125]
C. nubeculosus	Europe	BTV	Experimental transmission after oral infection	[128]
C. brevitarsis	Australasia	BTV	Experimental transmission after intrathoracic infection	[129]
C. brevitarsis	Australasia	BTV	Virus isolation ^d^	[130]
C. actoni	Australasia	BTV	Experimental transmission after oral infection	[131]
C. fulvus	Australasia	BTV	Experimental transmission after oral infection	[131]
C. wadai	Australasia	BTV	Experimental oral susceptibility	[131]
C. sonorensis	North America	EHDV	Experimental transmission after oral infection	[132]
C. insignis	Central America, Caribbean	EHDV	Experimental transmission after oral infection	[120]
C. imicola s.l.	Asia-Africa	EHDV	Virus recovery 10 days after oral infection	[133]
C. bolitinos	South Africa	EHDV	Virus recovery 10 days after oral infection	[133]
C. schultzei	Africa	EHDV	Virus isolation ^d^	[134]
C. oxystoma	Asia	EHDV	Virus isolation ^d^	[135]
C. brevitarsis	Australia	EHDV	Virus isolation ^d^	[136]
C. obsoletus	Europe	AHSV	Virus dissemination ^a^	[137]
C. imicola s.l.	Asia-Africa	AHSV	Virus dissemination ^a^	[87]
C. imicola	Asia-Africa	AHSV	PCR detection ^d^	[138]
C. sonorensis	North America	AHSV	Experimental transmission after oral infection	[139]
C. bolitinos	southern Africa	AHSV	Virus dissemination ^a^	[87]
C. bolitinos	southern Africa	AHSV	Virus isolation ^d^	[140]
C. scoticus	Europe	AHSV	Virus dissemination ^a^	[137]
C. obsoletus	Europe	SBV	PCR detection ^d^	[114,115]
C. obsoletus s.l.	Europe	SBV	PCR detection ^e^	[141]
C. scoticus	Europe	SBV	PCR detection ^d^	[115]
C. chiopterus	Europe	SBV	PCR detection ^d^	[142]
C. dewulfi	Europe	SBV	PCR detection ^d^	[113,114]
C. punctatus	Europe	SBV	PCR detection ^e^	[141]
C. paraensis	South America/Caribbean	OROV	Experimental transmission after oral infection	[27]
C. sonorensis	North America	OROV	Virus dissemination ^a^	[120]

^a^ suggesting transmission potential provided salivary gland barriers do not exist in *culicoids* (see Section 7), ^b^ independent demonstration of oral susceptibility and transmission after intrathoracic dissemination, ^c^ from unengorged, parous females, suggesting dissemination and transmission potential provided salivary gland barriers do not exist in culicoids (see Section 7), ^d^ from engorged complete females, not allowing conclusions on whether virus invaded haemocoel or remained in midgut, ^e^ from nulliparous females, suggesting dissemination, vertical transmission and transmission potential by bite provided salivary gland barriers do not exist in culicoids (see Section 7).

## Data Availability

No new data were created or analyzed in this study.
